# Functional and Nonfunctional Requirements of Virtual Clinic Mobile Applications: A Systematic Review

**DOI:** 10.1155/2024/7800321

**Published:** 2024-06-11

**Authors:** Zahra Parsaei, Majid Jangi, Shahram Tahmasebian, Asghar Ehteshami

**Affiliations:** ^1^ Health Information Technology Research Center Isfahan University of Medical Sciences, Isfahan, Iran; ^2^ Department of Medical Biotechnology School of Advanced Technologies Shahrekord University of Medical Science, Shahrekord, Iran

**Keywords:** mobile applications, self-care, telemedicine, virtual clinic

## Abstract

**Introduction:** The Virtual Clinic Mobile Application (VCMA) is a valuable tool for managing and remotely monitoring patients with various medical conditions. It can alleviate the strain on outpatient services and offer follow-up options for patients who do not require a physical examination. A thorough understanding of recent literature can assist in identifying suitable functionalities for new development and future improvement of current applications (apps). This review study is aimed at identifying functional and nonfunctional requirements for VCMA.

**Methods:** This study conducted a systematic search using databases such as PubMed, Scopus, ISI Web of Science, Science Direct, ProQuest, and IEEE to gather requirements of VCMA articles published in English from the inception of the databases up to April 2022. Out of a total of 1223 articles, 76 met the inclusion criteria. These articles were then analyzed using conventional content analysis to extract and categorize their requirements.

**Results:** Two main themes and 8 subthemes in terms of VCMA requirements were extracted as follows: (1) functional requirements with 3 subthemes (demographic data documentation, health record, general features of the user interface (UI)); (2) nonfunctional requirements with 5 subthemes (usability, accessibility, compatibility, efficiency, and security).

**Conclusion:** The findings highlight the importance of mHealth solutions for virtual care and the need for the development of apps based on the extracted functional and nonfunctional requirements for VCMA; however, controlled trials are necessary. It is recommended that transparent reporting of mHealth interventions be prioritized to enable effective interpretation of the extracted data.

## 1. Introduction

Regarding the increase in the patient population and global shortage of health professionals, timely access to healthcare, as one of the important challenges of service delivery, can be improved by the significant development of communication technology tools, especially smartphones, due to their ubiquitous nature [1–3].

From the beginning of the year 2023, more than 85% of individuals use smartphones and most of them have access to the Internet [4]. The growth of smartphone users fosters opportunity for professionals to replace conventional healthcare with Virtual Clinic Mobile Application (VCMA) [5].

Recently, VCMAs have been established as an acceptable tool for providing and receiving healthcare and are growing worldwide [6]. The virtual clinic allows a patient to communicate with a specialist through a multimodal contact, with the possibility of exchanging medical records, documents, and electronic prescriptions, when there is no need for a physical examination, before, during, and after the illness [7]. As to using a virtual clinic, there are several advantages including cost-effectiveness, access to treatment, prevention and early intervention over the phone or online, integration of necessary clinical pathways through communication between professionals, access to a network of physicians, reduction of appointments, and unnecessary encounter with medical centers [8].

Although the VCMA has a high potential for the prevention, diagnosis, follow-up, and treatment of patients, it reduces the pressure on the healthcare system and easily tackles the long-term needs of patients, but no study has been done to design and develop VCMA in such a way that the expected abilities of patients and specialists can be implemented. Therefore, this study endeavored to identify the functional and nonfunctional requirements of VCMAs.

The articles demonstrate the interest of doctors, paramedics, and patients in utilizing virtual communication options. However, most existing applications lack comprehensive virtual care capabilities, such as voice, video, and text calls with doctors. These applications are limited in scope, and there have been no efforts to design and develop a virtual clinic application that fully meets the expected requirements. Additionally, these applications are generally not endorsed by academic centers, lack scientifically validated design and development methods, and do not fully adhere to the scientific content approved by medical groups.

Therefore, the purpose of this research is to examine article reviews and their features that can be utilized to develop an application as a complementary tool to traditional care. This application is aimed at providing localized care, reducing travel expenses, and saving time in cases where physical examinations are unnecessary. Further studies are necessary to identify the factors that influence the acceptance of virtual clinics by patients, doctors, and paramedics.

## 2. Methods

In this review study, we extracted the functional and nonfunctional requirements of VCMAs by conducting a conventional content analysis of the apps.

### 2.1. Study Design and Search Strategy

To find relevant articles, a comprehensive electronic search was conducted on PubMed, Scopus, ISI Web of Science, Science Direct, ProQuest, and IEEE databases, from database inception up to April of the year 2022. The keywords “Mobile App∗” OR “Portable Software App∗” OR “Smartphone App∗” OR “Portable Electronic App∗” OR “Electronic App∗” AND “virtual clinic” OR “Online visit” OR “Online clinic” OR “virtual visit” OR “e visit” OR evisit OR “e clinic” OR clinic OR “electronic visit” OR “virtual care” OR “virtual healthcare” OR “virtual health care” OR “virtual outpatient clinic” OR “online outpatient clinic” OR “virtual ambulatory care facility” OR “outpatient online clinic” OR “online primary care visit” were searched for in the title/subject sections of the available articles. An Additional file 1 document is available for the search strategy applied in each of the databases. All articles obtained were then imported into EndNote software.

### 2.2. Inclusion Criteria

**Figure 1:**
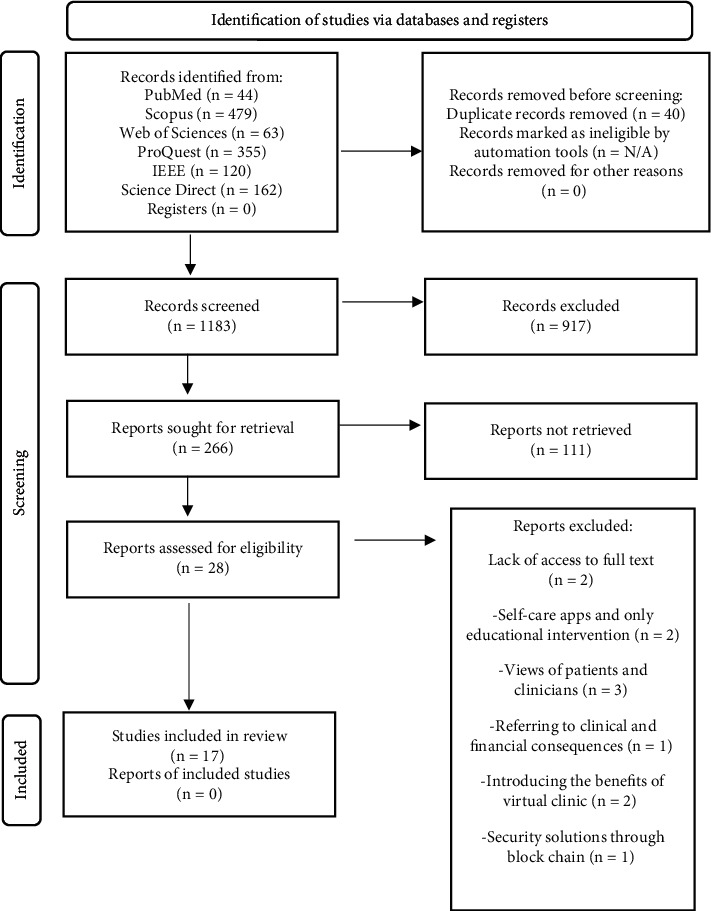
The process of database search and study selection.

The included studies have a) an app used to provide care through video call, phone call, or text; b) they have described the features or guidelines of VCMA and how to use it; and c) clinicians have participated in the design or validation of a VCMA. The excluded articles were like this: (1) lack of access to the full text (*n* = 2), (2) ones related to security solutions through blockchain (*n* = 1), (3) self-care apps and only educational interventions (*n* = 2), (4) referring to the clinical, financial and environmental consequences (*n* = 1), (5) the views of patients and clinicians (*n* = 3), and (6) introducing the benefits of the virtual clinic (*n* = 2). The process of database search and study selection are shown in Figure 1.

### 2.3. Study Selection

Two reviewers conducted a thorough screening of the studies based on the predetermined inclusion and exclusion criteria, considering both the title and abstract. Following this initial screening process, the two reviewers held virtual meetings to discuss the articles that met the criteria for inclusion. In the event of any discrepancies, a consensus was reached through comprehensive discussion.

### 2.4. Data Extraction and Synthesis

For each article, we initially extracted the following data independently using Conventional Content Analysis in MAXQDA 2020: author(s)' name, country, year of publication, methodology, app used, app purpose, sample size, duration of app usage, and features of VCMA (functional and nonfunctional requirements). In the second step, we categorized the functional and nonfunctional requirements and assigned titles to each group. In the third step, we refined, verified, and updated the titles, categorizations, and their relationships. Disagreements among researchers were resolved through discussion, review, and data reconciliation, resulting in an agreement.

### 2.5. Literature Quality Assessment and Risk of Bias Assessment

Researchers (AE and ZP) evaluated the quality of the articles using the Cochrane Collaboration's tool (Albornoz et al., 2022), which includes the following criteria: (1) random sequence generation, (2) allocation concealment, (3) blinding of participants and personnel, (4) blinding of outcome assessment, (5) incomplete outcome data, (6) selective reporting, and (7) other sources of bias. The results were divided into three groups: low risk of bias, high risk of bias, and unclear risk of bias. The interpretation of the quality assessment results based on guidelines was as follows: good (low risk for more than 2 items), fair (low risk for 2 items), or weak (low risk for less than 2 items).

## 3. Results

### 3.1. Search Results

The search results yielded 1223 studies from all six databases. EndNote's automatic duplicate function identified four duplicates, and an additional 36 duplicates were manually removed. By screening the titles and abstracts for relevant study objectives, 266 full texts were selected for further evaluation. After screening for relevant and sufficient information, 249 studies were excluded. Finally, after a thorough review of the full texts, 17 studies were included (Figure 1).

### 3.2. Characteristics of the Included Studies

The 17 studies included in this research were published in peer-reviewed journals. These studies were categorized as follows: three were literature reviews, three were pilot studies, two were proof-of-concept studies, one was a longitudinal observational study, one was an observational study, one was a retrospective observational study, one was a study protocol, one was a qualitative study, one was a cross-sectional study, one was an experiment with pre and posttest, one was descriptive, and one was a systematic review. The studies were conducted in a total of 10 different countries: five originated from the United States, three were from Canada, two were from China, and one each from Brazil, Australia, the Netherlands, Africa, Norway, India, and the United Kingdom (Additional file 2).

### 3.3. Functional and Nonfunctional Requirements

Functional requirements define the application's behavior in specific circumstances. They outline the programming tasks necessary to enable users to perform their work (user requirements) and ultimately meet the business requirements [9]. In the context of this study, functional requirements pertain to the functionalities that facilitate clinical services for patients. They are categorized into three main areas: (1) demographic data, (2) health record management, and (3) user interface (UI).

Nonfunctional requirements define how things should be done. They include features such as efficiency, security, functionality, compatibility, and applicability. Basically, they provide appropriate checks and comparisons for functional requirements [10]. The nonfunctional requirement in this study refers to the description of features that form aspects of software design and implementation. Based on the model presented in Aggarwal's article, these requirements are classified into five categories: (1) usability, (2) compatibility, (3) acceptability, (4) efficiency, and (5) security [11].

As shown in Tables 1 and 2, the extracted features of VCMA were categorized into two themes (functional and nonfunctional requirements) and eight subthemes. Those features that provide clinical service to the user were categorized as functional requirements (Table 1), and those that pertain to the design and implementation aspects of VCMA were categorized as nonfunctional requirements (Table 2).

## 4. Discussion

The engagement of patients, physicians, and paramedics is crucial when utilizing VCMA. Mobile applications have been developed globally in multiple languages to facilitate medical visits through synchronous and asynchronous phone, video, or text calls. However, Appireddy et al. discovered that physicians, paramedics, and patients prefer in-person visits for various reasons. These reasons include inadequate smartphone skills, limited access to high-speed Internet, concerns about privacy, differing pricing for visits, fear of encountering fake healthcare professionals or patients, and difficulty communicating effectively in a virtual setting [12]. Additionally, prominent physicians are hesitant to participate in electronic visits [13].

Kotecha et al. suggest that establishing standards for the development of virtual care and medical apps, based on expert opinions, will instill trust among customers and stakeholders, increase acceptance of these apps, and streamline insurance coverage for virtual services [14].

The findings of VCMA demonstrate that health records are vital for individuals to comprehend their necessary treatment and diligently adhere to their care plan. Furthermore, health records in VCMA facilitate data documentation monitoring and provide accurate information for clinicians to make more informed clinical decisions and diagnoses. These findings align with those of Armstrong et al., de Jong et al., and Dahne et al. [15–17]. Health records in VCMA also empower patients to enhance their self-awareness and engagement in self-care, promoting a sense of security, responsibility, and competence in self-management, as well as adherence to medications and prescriptions. Most patients can be relied upon to record such information and provide appropriate advice based on it [18, 19]. VCMA's online consultation should not be seen as a substitute for in-person visits. Instead, it serves as a supplemental option for outpatients who do not require physical examinations. It offers convenient access to healthcare providers through secure video calls and text messages [20]. According to Goldin et al., communicating with clinicians through text messages is beneficial as it reduces the amount of data exchanged in conversations by 50%. If patients are unable to convey their meaning through text, they can clarify it through a phone call [21]. Faruk et al. asserts that video and text communication are particularly advantageous for patients who prefer to avoid face-to-face interactions due to concerns about mental stigma and judgment [22]. Video visits are not only safe but also cost-effective as they eliminate the need for unnecessary travel and expenses [12]. Physicians have found video visits to be valuable and have noticed that patients are interested in using them for follow-up appointments [23]. Patients have reported that text, video, and audio calls allow for early treatment and prevention of further progression [24]. High-quality video calls enable specialists to gain a comprehensive understanding of patients' conditions [18]. Patients can conveniently access the desired service through VCMA by simply connecting to the internet. Knowing the scheduled visit time allows patients to plan ahead and effectively manage their time during the consultation [15, 19]. This helps to prevent any time wasted for both parties involved [18].

Patients have the option to select their preferred consultant by accessing the consultant's information in the VCMA and providing feedback on the consultant's performance, which aids in the decision-making process for other patients [25]. Conversely, consultants can improve patient behavior through feedback [19]. The selection of an appropriate specialist is paramount for patients with various conditions. For example, individuals with diabetes have discovered that text messages facilitate communication with their consultant, thereby improving their overall experience [15]. Patients using asynchronous text visits to quit smoking have reported finding them effortless and convenient, fostering a sense of trust and transparency in their care, which has motivated them to actively participate in their treatment [17]. Furthermore, patients who feel uneasy discussing their illness often find it more comfortable to communicate through text messages [19]. In the context of VCMA, reminders for appointments, medication administration, data documentation, and preventive care play a vital role, as both the patient and the therapist may overlook the appointment date and time [26]. The Graetz App has also demonstrated that medication reminders significantly enhance treatment adherence and alleviate related symptoms among breast cancer patients [27]. El Joueidi et al. further emphasized the utility of reminders for patients with chronic diseases [24]. Xu et al. also highlighted the significance of a reminder 15 min prior to the visit [18].

To enhance patients' knowledge about various diseases and expedite the treatment process, it is recommended to develop comprehensive training materials for each disease in VCMA. Armstrong suggests that these materials should be customized to address patients' specific needs, providing up-to-date information on treatment advancements and disease-related news and avoiding the use of generic training materials [15]. Goldin et al. implemented a feature called “My Page” in his app, which offered easy access to training materials and proved effective in reducing symptoms of depression [21].

Engaging in chat room discussions with therapists can help patients expand their understanding of diagnoses, treatments, and prevention of complications. These conversations also assist in making decisions regarding necessary visits, while facilitating information sharing, addressing concerns, and seeking advice from peers who are in similar situations [28]. As a result, chat rooms play a crucial role in self-management [15]. Goldin et al. discovered that group chats among patients with depression, their peers, and therapists created a safe space where patients could exchange opinions without fear of judgment [21]. Cruz et al.'s research on VCMA revealed that chat rooms helped patients with breast cancer acquire knowledge about their treatment process and potential complications, enabling them to better cope with their illness [29]. Additionally, Couturier et al.'s findings demonstrated that virtual chats for patients with eating disorders, conducted in a supportive environment, were valuable for receiving emotional support and preventing disease relapse [30].

The user manual is an integral part of VCMA. Goldin et al. provided comprehensive training for online visits through videos, text, recorded sound, and graphic images. He also designed a page containing information on app usage conditions and regulations, ensuring users can utilize the service without any concerns or difficulties [21]. Similarly, Burton also provided necessary training for VCMA users [19].

Patients and clinicians can easily share various types of media, such as images, videos, audio, and radiographs, through the information-sharing feature. In Burton et al.'s study, physicians found that most of the patients' information was both helpful and efficient [19].

Since the users of VCMA come from diverse backgrounds with varying levels of literacy and social class, it is important to consider different aspects of the UI in a clear and unambiguous manner. The usability and technical aspects of the UI play a significant role as they serve as the main point of interaction between the user and the app, ultimately improving its usability. A well-designed UI for VCMA enables users to quickly find the necessary information, reducing the time spent on searching. Therefore, in addition to functional requirements, adequate attention should be given to nonfunctional requirements during the UI design process. These include the user experience of VCMA, verifying physicians by validating their medical council number and educational documents during registration, carefully evaluating instructions and training videos, the ability to perform advanced searches in multiple languages, compatibility with various operating systems, adherence to aesthetic principles, ensuring security, privacy, and accuracy, as well as developing a multilingual UI for the international expansion of specific VCMA services.

This research has a few limitations. First, we excluded articles published after April 2022 from this review. Second, we solely focused on reviewing articles and did not evaluate applications available in markets such as Google Play and the App Store.

## 5. Conclusions

Articles demonstrate the interest of physicians, paramedics, and patients in utilizing virtual options for communication. The VCMA model, developed based on literature, past experiences, and future needs, offers effective support for clinical practice and patient care in a virtual clinic setting. It facilitates online consultations and remote medical advice. The VCMA also enables users to schedule appointments, access their medical records, get prescription refills, and receive reminders for medication or follow-up appointments. Moreover, it provides secure messaging for communication with physicians and allows users to search for specific medical information.

Therefore, it is recommended that application developers in developing countries take the following factors into account when developing such applications:

(1) Identify the target audience and their specific needs. (2) Understand the cultural and social contexts in which the application will be used. (3) Assess the infrastructure and technological capabilities of the country. (4) Consider the regulatory and legal frameworks that may impact the application's deployment and usage. (5) Evaluate the economic conditions and purchasing power of potential users. (6) Analyze the competition and existing market landscape in the country. By considering these factors, developers can ensure that their applications are tailored to the specific context and needs of the developing country, resulting in increased user adoption and success.

## Figures and Tables

**Table 1:** Functional requirements of VCMA.

**Requirements**		**Descriptions**
A. Demographic data documentation		• Identity: first name and surname • Insurance: insurance name and number

B. Health record	Vital sign documentation	• Blood pressure • Heart rate
	Kidney documentation	• Blood sugar
	Fitness documentation	• Weight • Exercise • Sleep • Nutrition
	Skin documentation	• Condition of skin
	History documentation	• Disease history • Family history • Allergy • Medications history • Vaccination • Surgeries • Smoking
	Present history documentation	• Symptoms and conditions • X-ray • Tests • Treatments • Prescriptions • Patient training
	Documentation of inpatient treatment history	• Date of admission and discharge • Hospital name • Chief complaint • Summary
	Medication documentation	• Name • Dose • Type • Instruction • Side effects

C. General features of UI	Reminders	• Appointment • Preventive care
	Display	• Patient's self-declaration about his health status • Consultant identity: name and surname and profile picture • Curriculum vitae: expertise and consulting field • Type of communication: phone call, text, video call, and real-time • Appointment: consultation date, time, duration, and waiting time for response
	Service selection	• Choosing consultation day, time, and type of insurance
	Reporting	• Providing weekly/monthly reports on the patient's treatment process • Providing weekly/monthly reports of vital signs and medical history
	Support	• Documentation of communication ways: tagging capability in VCMA and frequently asked questions • Q and A ability: discussion forum with others (patients and consultants)
	Help/tutorial	• Text guide: help • Training inside VCMA: working with it • About us • Terms and conditions of use

**Table 2:** Nonfunctional requirements of VCMA.

**Requirements**		**Descriptions**
A. Usability	Setting	• Creating a personal profile for the user (consultant-patient) • Microphone • Camera • Measurement units selection: selection of measurement units like centimeters and meters for height • Video call with other participants: making a video call with other connected individuals • Selecting conversation starter • Log out

B. Accessibility	Service	• Save and mark: appointments, reminders, notes, recorded voices, photos, and messages; send feedback on consultant's performance and select text from a default list • Ability to share: individual lessons, health record information with doctors, and patient-consultant data exchange

C. Compatibility	Service format	• Insurance: recording name and number

D. Efficiency	Service	• Financial transaction reporting

E. Security		• Login method: text password, email, activation code, and anonymously • Verify the user's identity by sending a verification code to the mobile phone • Possibility of deleting consultation records, documents, and bank transactions • Encrypted patient data exchange

## Data Availability

The datasets used and/or analyzed during the current study are available from the corresponding author upon reasonable request.

## Supporting Information

Supporting Information 1 Search strategy.

Supporting Information 2 Characteristics of the included studies.

Supporting Information 3 PRISMA 2020 Checklist.
