# Supplementary material for: Functional and Nonfunctional Requirements of Virtual Clinic Mobile Applications: A Systematic Review
Source: Int J Telemed Appl. 2024 Jun 11;2024:7800321. doi: 10.1155/2024/7800321 (PMC11186682; doi:10.1155/2024/7800321)
Supplement: Supporting Information 1 — Search strategy. [file 7800321.f1.docx]

**Search Strategy**

| **Data of Search** | **Results** | **Search line** | **Database** |
| --- | --- | --- | --- |
| 2022/04/4 | 44 | ("Mobile Applications"[Mesh]OR Application, Mobile OR Applications, Mobile OR Mobile Application OR Mobile Apps OR App, Mobile OR Apps, Mobile OR Mobile App OR Portable Software Apps OR Software App, Portable OR Portable Software Applications OR Application, Portable Software OR Portable Software Application OR Software Application, Portable OR Smartphone Apps OR App, Smartphone OR Apps, Smartphone OR Smartphone App OR Portable Electronic Apps OR App, Portable Electronic OR App, Portable Electronic OR App, Portable Electronic OR Electronic App, Portable OR Portable Electronic App OR Application, Portable Electronic OR Portable Electronic Applications OR Electronic Application, Portable OR Portable Electronic Application) AND (((((((((((((((("virtual clinic "[Title/Abstract]) OR ("Online visit "[Title/Abstract])) OR ("Online clinic"[Title/Abstract])) OR ("virtual visit "[Title/Abstract])) OR ("e-visit"[Title/Abstract])) OR ("evisit"[Title/Abstract])) OR ("e visit"[Title/Abstract])) OR ("e-clinic"[Title/Abstract])) OR ("eclinic"[Title/Abstract])) OR ("e clinic"[Title/Abstract])) OR ("electronic visit"[Title/Abstract])) OR ("virtual care"[Title/Abstract])) OR ("virtual healthcare"[Title/Abstract])) OR ("virtual outpatient clinic"[Title/Abstract])) OR ("online outpatient clinic"[Title/Abstract]))) | ***PubMed*** |
| 2022/04/5 | 479 | TITLE-ABS ( "Mobile Application*" OR mobile AND app* OR portable AND software AND app* OR smartphone AND app* OR portable AND electronic AND app* ) AND TITLE-ABS ( "online clinic" OR "virtual clinic" OR "virtual visit" OR "e visit" OR "e-visit" OR "e clinic" OR "e-clinic" OR "electronic" OR "virtual care" OR "virtual health care" OR "virtual ambulatory care facility" OR "virtual outpatient clinic" OR "online outpatient clinic" OR "outpatient online clinic" OR "online primary care visit" OR "online outpatient care" ) AND ( EXCLUDE ( PUBYEAR , 2023 ) ) AND ( LIMIT-TO ( LANGUAGE , "English" ) OR LIMIT-TO ( LANGUAGE , "Persian" ) ) AND ( LIMIT-TO ( DOCTYPE , "ar" ) OR LIMIT-TO ( DOCTYPE , "ch" ) OR LIMIT-TO ( DOCTYPE , "re" ) OR LIMIT-TO ( DOCTYPE , "bk" ) ) | ***Scopus*** |
| 2022/04/30 | 63 | Mobile App OR App*, Mobile OR Portable Software App* OR Smartphone App* OR Portable Electronic App* (Topic) and "virtual clinic" OR "online visit" OR "online clinic" OR "virtual visit" OR "evisits" OR "e-visit" OR "e-clinic" OR "eclinic" OR "electronic visit" OR "virtual care" OR "virtual healthcare" OR "virtual ambulatory care facility" OR "virtual outpatient clinic" OR "online outpatient clinic" OR "outpatient online clinic" OR "online primary care visit" OR "online outpatient care" (Topic) \| | ***ISI Web of Sciences*** |
| 2022/04/06 | 355 | TI(online clinic OR virtual clinic OR e-visit OR virtual visit OR e visit OR e clinic OR e-clinic OR virtual care OR virtual health care OR virtual ambulatory care facility OR virtual outpatient clinic OR online outpatient clinic OR outpatient online clinic OR online primary care visit OR online outpatient care) NOT (at.exact("Commentary" OR "Correspondence" OR "Conference Proceeding" OR "Report" OR "Correction/Retraction" OR "News" OR "Letter to the Editor") AND la.exact("ENG") AND pd(20040101-20220406)) | ***ProQuest*** |
| 2022/04/05 | 120 | (("All Metadata":"Mobile Application*" OR "All Metadata":Mobile App* OR "All Metadata":Portable Electronic App* OR "All Metadata":Portable Software App* OR "All Metadata": Smartphone App*) AND ("All Metadata":online clinic) OR ("All Metadata": electronic visit) OR ("All Metadata":virtual clinic) OR ("All Metadata":e-visit) OR ("All Metadata":virtual visit) OR ("All Metadata":e-clinic) OR ("All Metadata": virtual health care) AND ("All Metadata":virtual care) )  Filters Applied: 1981 - 2022Open Access Only | ***IEEE*** |
| 2022/04/07 | 162 | ("Mobile App" OR Software OR Smartphone App) AND (online clinic OR virtual clinic OR virtual visit OR e visit OR e clinic OR virtual health care) | ***Science Direct*** |
