# Supplementary material for: Functional and Nonfunctional Requirements of Virtual Clinic Mobile Applications: A Systematic Review
Source: Int J Telemed Appl. 2024 Jun 11;2024:7800321. doi: 10.1155/2024/7800321 (PMC11186682; doi:10.1155/2024/7800321)
Supplement: Supporting Information 2 — Characteristics of the included studies. [file 7800321.f2.docx]

**Characteristics of the included studies**

|  | **Authors/ Country** | **Methodology** | **App used** | **App purpose** | **Features of the**  **Mobile application** | **Sample** | **Duration of**  **mobile app**  **usage** |
| --- | --- | --- | --- | --- | --- | --- | --- |
| 1 | Dahne et al  2020  United states([9](#_ENREF_9)) | pilot trial | MY chart | The effect of using electronic visits on smoking cessation | - Registration of medication instructions - Smoking history - E-visit - Video call - Record Test result | 51 | 1-3month |
| 2 | Goldin et al  2019  United states([10](#_ENREF_10)) | Longitudinal Observational Study | Ascend Program | The effect of group chat on the treatment of Depression | - Support users through text in the app - text &video call - Patient education - Viewing the writings of others - Notifications - Emergency Plan - Privacy Policy - Terms of Use | 117 adults with elevated depressive symptoms | 4weeks |
| 3 | Melcher et al  2021  United states([11](#_ENREF_11)) | observational study | mindLAMP app | collect active and passive data on users and to serve as a mental health care management platform. | - Login with activation code - The ability to delete information by the user - Video call | 100 college students | 28Days |
| 4 | Kunkle & Hunt.  2020  United states([12](#_ENREF_12)) | Retrospective Observational Study | Ginger mobile app | reducing depression symptoms | - Terms of Use - Login with access code - text and video call - select text from the default list - about us - Information of the consultant (name and last name, field of consultation, time and day of consultation) - create personal profile (patient and consultant) | 1662 users | 8-12 week |
| 5 | Mallow et al  2015  United states([13](#_ENREF_13)) | Study protocol | mI SMART | Providing service to rural and low-income areas | - Login with username and password - text and video call - Record blood pressure - weight - glucose - education about blood glucose monitoring, medication, nutrition, exercise, foot care, heart disease, complications of chronic illnesses, and behavior change. - Record the health history, medication and treatments | adults aged 18–64(30 participants) | 12 weeks |
| 6 | Couturier et al  2021  Canada([14](#_ENREF_14)) | scoping review | Guideline | developing high quality treatment guidelines that are focused on virtual interventions for children, adolescents, and  emerging adults with eating disorders, and their caregivers | - Chat with other peers - Making text and video calls with consultants | children, adolescents, and emerging adults with eating disorders, and their caregivers. | 1-14weehs |
| 7 | El Joueidi et al  2021  Canada & South Africa([15](#_ENREF_15)) | cross-sectional | Wel Tel | WelTel has been implemented for HIV, asthma, and tuberculosis care, and in rural areas to support primary care clinics. | - Short message service (SMS) - voice and video call - Appointment scheduling and reminders - data sharing - Prescription - Record symptoms - treatments and Vaccination | 138 | 17-months |
| 8 | Hwang  2016  Canada([16](#_ENREF_16)) | Experiment with pre and after the test | Medeo | Investigating the effectiveness of the Medeo application  To reduce face-to-face visits | - Login to the app with email and password - Sending text messages and - attaching files and photos | 72 patients (35 people in case group and 37 people in  control group) | 3week |
| 9 | Li et al  2019  China([17](#_ENREF_17)) | pilot study | WeChat software (Tencent, Shenzhen, China) | Providing service to vascular patients during Covid-19 | - Video calling with the ability to connect family members - Store and mark treatments - Video call recording | 114 vascular patients | 11mint |
| 10 | Xu et al  2021  China([18](#_ENREF_18)) | pilot study | PD CARE | Receive post-surgery care | - Prescribe - Set appointment time - Registration of medication information - fitness: exercise, sleep and meal - Patient’s self-statement about their disease process - Password and ID card to enter the app - Video calls - Reminder to patients 15 minutes before the meeting and initiated the video call - Real time online consultation | 22Parkinson’s disease patient | 4weeks |
| 11 | Hasselgren et al  2021  Norway([19](#_ENREF_19)) | Proof-of-Concept Study | Guideline | Creating the right infrastructure and encryption for virtualization | Verification of caregiver credentials, Treatment information is sent in code | NA | NA |
| 12 | Kho et al  2020  Australia([20](#_ENREF_20)) | Review | MTD | Identifying the characteristics of apps in the skin field | Sharing a photo, text &video call, User authentication, Anonymous membership | mobile teledermoscopy | NA |
| 13 | Jong  2018  Netherlands([21](#_ENREF_21)) | Proof-of-Concept Study | 1. Kanta  2. Facetalk  3. MedApp | assessing the feasibility and functionality of a new cloud-based and multicomponent outpatient  clinic, the “Virtual Outpatient Clinic” (VOC). | - Record blood pressure, weight - Text, phone & video call - Registration of drugs used - notification | 10 patients from both Departments of Internal Medicine and Dermatology (N=20) | 6week |
| 14 | Faruk et al  2017  Africa ([22](#_ENREF_22)) | Descriptive | Guideline | Providing a solution for the successful implementation of the virtual clinic | - Security and privacy (sending encrypted data) - Sharing radiology images, audio - Record health history - Insurance - List of drugs used |  |  |
| 15 | Cruz et al  2019  Brazil([23](#_ENREF_23)) | Systematic Review | Owise  BCS  Health Weaver Mobile | Providing virtual services to cancer patients | - Medication reminder - Sharing health information with the therapist - Online counseling - Health diary - Login with username and password - Settings - about us - Terms of Use - Save and bookmark: appointments, notes, messages, voice recordings, photos - List of questions | 18 studies |  |
| 16 | Burton et al  2020.  United Kingdom([24](#_ENREF_24)) | Qualitative Study | micro practice care model | Providing virtual medical service during the covid-19 pandemic for rural patients | - Video, text and phone calls - Save reminder - The ability to select the initiator of the connection - Frequently Asked Questions - Learning how to work with the software - Feedback from Caregiver performance - Registration of vital signs (blood pressure, heart rate) - Weight - blood glucose - Sharing information with the caregiver | 8 patients from a  rural micro practice | NA |
| 17 | Appireddy et al  2020  India([25](#_ENREF_25)) | Review | Facetime WhatsApp, Zoom, Skype, or Google Duo | overview of the virtual care, relevance to neurology and  some guidance on implementing virtual care in an Indian context. | - Video call | NA | NA |
